# Age-stratified prevalence and associated risk factors of hypertension, diabetes and hypercholesterolemia in Nepal: Insights from the STEPS survey 2019

**DOI:** 10.1371/journal.pone.0340932

**Published:** 2026-10-16

**Authors:** Durga Datta Chapagain, Kennedy Mensah Osei, Iga Danik Prasiska, Vasuki Rajaguru, Sunjoo Kang, Heejin Kimm, Tae Hyun Kim, Sang Gyu Lee, Whiejong Han

**Affiliations:** 1 Global Health Security, Graduate School of Public Health, Yonsei University, Seoul, South Korea; 2 Ministry of Health, Bagamati Province, Nepal; 3 Healthcare Management, Graduate School of Public Health, Yonsei University, Seoul, South Korea; 4 Department of Global Health and Disease control, Graduate School of Public Health, Yonsei University, Seoul, South Korea; 5 Institute for Health Promotion, Graduate School of Public Health, Yonsei University, Seoul, Republic of Korea; 6 Department of Preventive medicine, College of Medicine, Yonsei University, Seoul, South Korea; Atal Bihari Vajpayee Institute of Medical Sciences & Dr Ram Manohar Lohia Hospital, INDIA

## Abstract

**Background:**

Non-communicable diseases (NCDs) are a major global public health challenge, particularly in low- and middle-income countries such as Nepal. Despite the growing burden of hypertension, diabetes, and hypercholesterolemia, limited evidence exists on age-stratified patterns of NCDs and associated risk factors, especially among younger populations. This study investigated age-stratified prevalence of hypertension, diabetes and hypercholesterolemia and risk factors in Nepal to inform age-specific preventions and policy interventions.

**Methods:**

We analyzed nationally representative data from the 2019 STEPS Survey, including 3,219 participants aged 15–69 years. Descriptive statistics and chi-square tests describe participant characteristics. Multivariable logistic regression assessed associations between risk factors and NCDs, while univariate logistic regression, Wilcoxon scores, and pairwise multiple comparisons examined age-group differences in prevalence of NCDs and risk factors.

**Results:**

The mean participant age was 35.3 ± 12.8 years, with 56% female. Among adults aged ≥60 years, 50% used tobacco and 31.5% consumed alcohol. Alcohol consumption was also notably prevalent among individuals aged 15–29 years, affecting 30.7% of this age group. Physical inactivity was identified in 18.9% of the overall study population. The highest rates of overweight (30.2%) and stress (80.1%) were among adults aged 30–44, with obesity (9.0%) peaking at 45–59 years. Men (OR 2.04, 95% CI 1.62–2.55) and those aged 60 years or older (OR 4.92, 95% CI 3.35–7.22) had higher odds of hypertension. Diabetes (OR 6.34, 95% CI 3.58–11.23) and hypercholesterolemia (OR 2.66, 95% CI 1.77–3.98) were most prevalent in the 45–59 age group.

**Conclusions:**

The prevalence of NCDs and risk factors increases with age, yet substantial burdens exist among younger adults. Age-specific targeted policies should promote healthy behaviors and routine screening in youth, emphasize metabolic risk management and early detection in middle age, and ensure sustained treatment access and continuity of care for older adults.

## Introduction

Non-communicable diseases (NCDs) burden has evolved into a global public health emergency that needs attention and appropriate interventions for prevention and control [1]. Globally, NCDs account for approximately 41 million deaths annually, representing nearly 74% of all deaths worldwide, with 17 million annual deaths before age 70, about 86% premature NCD related deaths occur in low-and-middle-income countries (LMIC). Over 80% of these premature deaths are due to cardiovascular diseases (CVD), cancers, chronic respiratory diseases and diabetes [2]. In Nepal, NCDs cause 66% of deaths: 30% from CVD, 10% from chronic respiratory conditions, 9% from cancer, 4% from diabetes, and 13% from others [3]. The global rise in NCDs is associated with lifestyle factors: unhealthy diet, physical inactivity, harmful alcohol and tobacco use, obesity, high blood pressure, and elevated cholesterol and blood glucose levels; similar trend seen in Nepal [2,4]. Early onset of these risk factors contributes substantially to long-term morbidity, disability, healthcare expenditure, reduced productivity, and premature mortality, particularly in LMICs with limited healthcare resources.

The growing burden of NCDs has become a major global health and development concern, reflected in the Sustainable Development Goal (SDG) target 3.4, which aims to reduce premature mortality from NCDs by one-third through prevention and treatment by 2030 [5]. In response, the World Health Organization developed the WHO Global Action Plan for the Prevention and Control of Noncommunicable Diseases 2013–2020 to guide countries in strengthening NCD prevention, surveillance, early detection, and management through multisectoral approaches [6]. Nepal has also implemented the Multisectoral Action Plan for the Prevention and Control of Noncommunicable Diseases to address the increasing burden of NCDs through coordinated strategies focusing on health promotion, risk-factor reduction, early diagnosis, and strengthening primary healthcare systems [7]. Despite these initiatives, implementation challenges remain, particularly in achieving effective age-specific prevention, early detection, and long-term management of NCDs.

Several studies have consistently shown that NCDs are common in older adults [8–11]. Nevertheless, in LMIC, younger people often develop NCDs, resulting in considerable social, economic and health consequences. In 2016, an estimated 1.7 million (4%) NCD deaths were aged below 30 years, 15.2 million (38%) aged 30–70, and 23.6 million (58%) were aged above 70 years [12]. Despite this growing burden, most of the NCDs prevention initiatives focus on older adults, overlooking the burden among young adults. The common perception that young individuals are generally healthy and less vulnerable to chronic diseases may contribute to delayed prevention and early detection efforts among younger age groups. Nevertheless, young adults bear a significant part of global NCD burden due to rising rates of obesity, physical inactivity, tobacco use, alcohol consumption, and unhealthy dietary patterns [13,14]. Understanding age-stratified patterns of NCD prevalence and associated risk factors is therefore essential for effective health policy planning, resource allocation, targeted prevention strategies, and early intervention programs.

Although several studies in Nepal have reported the overall prevalence of NCDs and their associated risk factors, limited evidence exists regarding age-stratified differences in Hypertension, Diabetes and Hypercholesterolemia; and related behavioral and metabolic risk factors using nationally representative data. Furthermore, previous studies have rarely examined how sociodemographic, lifestyle, and clinical risk factors vary across age groups, limiting the development of age-specific interventions and policies. Addressing this evidence gap is important for strengthening NCD prevention and control strategies in Nepal.

The World Health Organization (WHO) STEP-wise approach to surveillance (STEPS) is a standardized, internationally comparable framework designed for monitoring the risk factors of NCDs such as cardiovascular diseases, diabetes, and cancer. The STEPS survey is implemented in three stages: Step 1 gathers sociodemographic and behavioral data (including tobacco and alcohol use, diet, and physical activity); Step 2 collects physical measurements (including height, weight, blood pressure, and waist circumference); and Step 3 involves biochemical assessments (including blood glucose, cholesterol, and triglycerides). This modular design allows countries to systematically collect, analyze, and disseminate key NCD risk factor data to inform health policies and programs.

In Nepal, the STEPS survey was conducted between February and May 2019 as a nationally representative cross-sectional study, targeting adults aged 15–69 years from all seven provinces through a multistage cluster sampling method. The survey included 6,475 eligible participants and assessed both behavioral and biological risk factors for NCDs. Data were collected using the WHO STEPS instrument version 3.2, with additional modules on topics such as oral health, mental health, injury, violence, and cervical cancer screening. The survey provides comprehensive insight into the distribution and determinants of NCD risk factors in Nepal, a country where about two-thirds of annual deaths are attributable to NCDs.

Therefore, this study aimed to comprehensively assess the prevalence of diabetes, hypertension, and hypercholesterolemia, along with their associated risk factors, across stratified age groups using nationally representative data from the 2019 STEPS survey in Nepal. Additionally, the study aimed to identify sociodemographic, behavioral, and clinical risk factors associated with these conditions and provide evidence-based recommendations for age-specific prevention strategies, early screening, and policy interventions to strengthen NCD prevention and control efforts in Nepal.

## Methods and materials

### Study design and study population

This observational cross-sectional study utilized nationally representative data from the 2019 Nepal STEPS Survey, which employed the standardized and customized WHO STEPS tools [15]. The data were obtained from the WHO NCD Microdata Repository [16]. The WHO-STEPS approach is a comprehensive platform for countries to collect, analyze, and disseminate standardized NCD risk factors information [17]. The STEPS Survey 2019 was conducted across all seven provinces of Nepal using a multistage cluster sampling design. Primary sampling units (clusters) were selected using probability proportional to size sampling, followed by systematic household selection and random selection of eligible participants aged 15–69 years within households. Data was collected from 25 participants in each cluster, requiring approximately 36–37 clusters per province, resulting in a total of 259 clusters nationwide. Within each province, the number of clusters was allocated across metropolitan, sub-metropolitan, municipal, and rural municipal sub-strata in proportion to their share of the total provincial population. Data was collected through face-to-face interviews and physical/biomedical measurements [16].

Participants aged 15–69 years who had resided in the household for at least six months and were present the night before the survey were included. Participants with missing data on key variables were excluded from the analysis. Of the 6,475 eligible participants in the national survey, 5,593 completed the core survey. Following complete-case analysis, 3,219 participants were included in the final analysis for this study.

The STEPS survey had previously obtained ethical approval from the relevant authorities for data collection. Official permission to use the *Nepal STEPS Survey 2019* dataset for this study was granted by the World Health Organization NCD Microdata Repository on 14 November 2023. The dataset is publicly accessible through the WHO NCD Microdata Repository and the WHO NCD surveillance website (study data available at https://extranet.who.int/ncdsmicrodata/index.php/catalog/771 and the survey report available at https://www.who.int/teams/noncommunicable-diseases/surveillance/data/Nepal; Accessed on 14 November 2023). See also S1 File**.** for the dataset used in this study and the survey report.

### Variables

Prevalence of NCDs: diabetes, hypertension and hypercholesterolemia were taken as dependent variables. Fasting blood glucose was assessed on-site using a glucometer. Individuals with levels >125 mg/dL or undergoing diabetes treatment were classified as diabetic. Blood pressure was measured three times at ≥15-minute intervals using a digital sphygmomanometer. Hypertension was defined as average systolic ≥140 mmHg, diastolic ≥90 mmHg, or current use of antihypertensive medication. Venous blood samples were collected for serum total cholesterol analysis. Hypercholesterolemia was defined as total cholesterol >200 mg/dL or current treatment for the condition. Age was the main explanatory variable, categorized into 4 groups: young adults (15–29 years), lower-middle adults (30–44 years), upper-middle adults (45–59 years), and elder (≥60 years). Independent variables included sociodemographic factors (sex, education, ethnicity, marital status, occupation, family size, wealth quintile, region and residence), behavioral/lifestyle factors (tobacco use, alcohol consumption, physical inactivity and stress) and biomedical/physical measurements (BMI). Sociodemographic and behavioral/lifestyle data were collected via oral interviews. Body Mass Index (BMI) was calculated as weight in kilograms divided by height in meters squared (kg/m^2^), following WHO guidelines [18]. The education level was categorized as below secondary level, secondary level and higher level. The ethnicity was categorized into 4-groups: Upper caste (Brahmin/Chhetri), Disadvantaged (Janajati/non-Dalit Terai), Relatively Advantaged Janajati, and Others (Dalit/Religious Minorities/Others). The occupation was categorized as unemployed, employed (government/non-government), Self-employed, homemakers and students. The seven provinces were categorized into 4-regions by geography and Human Development Index (HDI) [19]: East (Koshi: HDI = 0.58), Middle (Bagamati/Gandaki: HDI = 0.661–0.618), South (Madhesh/Lumbini: Mostly Plane area and HDI = 0.510–0.563) and West (Karnali/Sudur Paschim: Mostly hilly area and HDI = 0.538–0.547). BMI was categorized as underweight (≤18.5), normal (18.5–24.9), overweight (25–29.9) and obese (≥ 30) according to WHO guidelines [18]. Fig 1 presents the structured study design, and S1 Table. contains the operational definitions.

**Fig 1:**
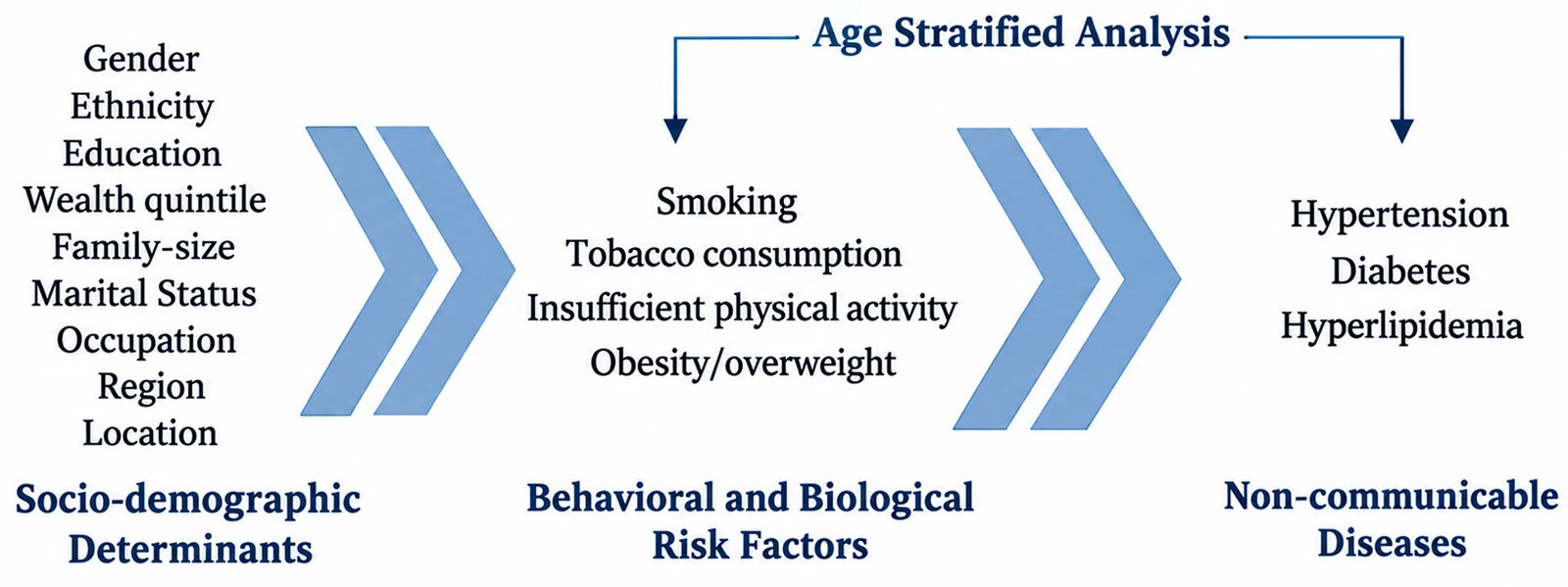
Conceptual Framework: Age-stratified Analysis of NCDs and Associated Factors in Nepal.

### Statistical analysis

Descriptive statistics were used to summarize the sociodemographic, behavioral, and clinical characteristics of participants across age groups. Continuous variables were presented as mean ± standard deviation (SD), while categorical variables were expressed as frequencies and percentages. Chi-square tests were performed to assess differences in categorical variables among age groups.

Multivariable logistic regression analyses were conducted to identify factors associated with hypertension, diabetes, and hypercholesterolemia after adjusting for potential confounders. Age group, sex, education, ethnicity, marital status, occupation, family size, household wealth quintile, region, residence, tobacco use, alcohol consumption, physical activity, stress, and body mass index (BMI) were included as explanatory variables in the regression models based on epidemiological relevance and previous literature. The goodness of fit of the multivariable logistic regression models was assessed using the Hosmer–Lemeshow goodness-of-fit test. A non-significant Hosmer–Lemeshow test (*p* > 0.05) was considered indicative of no evidence of poor model fit. Nagelkerke R^2^ was reported as a pseudo-R^2^ measure to describe the explanatory power of each model. The likelihood ratio test and Akaike information criterion (AIC) were also used as supplementary measures of model fit and model comparison. Multicollinearity among explanatory variables was assessed using tolerance and variance inflation factor (VIF) statistics. No significant multicollinearity was detected, as all VIF values were below 5 and tolerance values exceeded 0.1. The highest VIF observed was 4.20 among occupation-related variables in the hypercholesterolemia regression model, indicating acceptable collinearity among predictors. Adjusted odds ratios (AORs) with 95% confidence intervals (CIs) were reported. Age-stratified associations between age groups and NCD outcomes were additionally examined using unadjusted logistic regression models.

Normality assessment using PROC UNIVARIATE and visual inspection indicated slight skewness in age distribution. However, age was categorized into four age groups (15–29, 30–44, 45–59 ans ≥ 60. For the rank-based comparison of hypertension, diabetes, and hypercholesterolemia across age groups, Wilcoxon rank scores were calculated, with average ranks assigned to tied observations. Overall differences across the four age groups were assessed using the Kruskal–Wallis test. When the overall Kruskal–Wallis test was statistically significant, pairwise comparisons were conducted using the Dwass–Steel–Critchlow–Fligner (DSCF) method based on Wilcoxon rank sums. Receiver operating characteristic (ROC) curve analyses were used to assess the discriminatory ability of age in predicting hypertension, diabetes, and hypercholesterolemia. All statistical tests were two-tailed, and statistical significance was considered at p < 0.05. All statistical analyses were conducted using SAS version 9.4 (SAS Institute Inc., Cary, NC, USA).

## Result

A total of 3,219 participants with complete data on education, BMI, diabetes, and hypercholesterolemia variables were included in the analyses after excluding missing observations from the original STEPS Survey sample (Fig 2).

**Fig 2:**
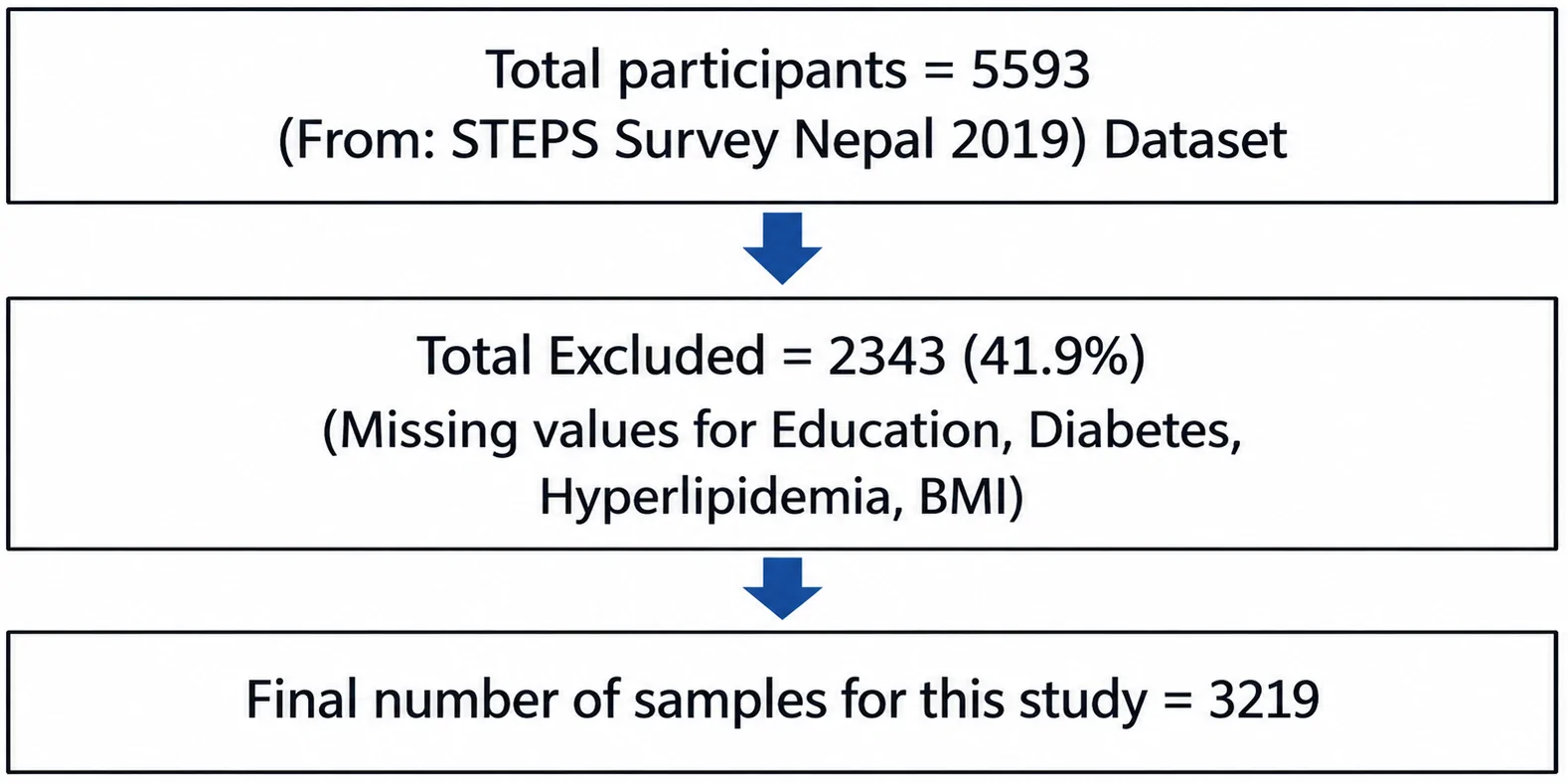
Sample selection flow chart.

### Participants’ characteristics by age group

Table 1 presents the sociodemographic, behavioral, biomedical, and clinical characteristics of the study population stratified by age groups. The mean age of participants was 35.3 ± 12.8 years, and 56.1% were female. The largest proportion of participants belonged to the 30–44 years age group (38.8%). The proportion of participants with less than secondary education increased in older age groups. Most participants had less than secondary education (49.1%), were currently married (83.3%), belonged to upper caste ethnic groups (42.2%), lived in urban areas (64.8%), and were homemakers (45.0%).

**Table 1:** Sociodemographic, behavioral, biomedical, and clinical characteristics by age group.

Variables	Categories	Age Group (years) (N = 3219)					
		Total	15-29	30-44	45-59	≥60	p-value
		N (%)	N (%)	N (%)	N (%)	N (%)	
**Age** (Mean ± SD = 35.3 ± 12.8)		3219	1191 (37)	1249 (38.8)	601 (18.7)	178 (5.5)	
**Sex**	Female	1805 (56.1)	792 (65.5)	758 (60.7)	214 (35.6)	41 (23)	<0.001
	Male	1414 (43.9)	399 (33.5)	491 (39.3)	387 (64.4)	137 (77)	
**Education**	Less than secondary	1582 (49.1)	416 (34.9)	657 (41.5)	371 (61.7)	138 (77.5)	<0.001
	Secondary	1473 (45.8)	715 (60)	528 (42.3)	196 (32.6)	34 (19.1)	
	Above secondary	164 (5.1)	60 (5)	64 (5.1)	34 (5.7)	6 (3.4)	
**Ethnicity**	Upper caste	1358 (42.2)	460 (38.6)	525 (42)	293 (48.7)	80 (44.9)	<0.001
	Disadvantaged (Janajati/non-Dalit Terai)	829 (25.7)	319 (26.8)	334 (26.7)	139 (23.1)	37 (20.8)	
	Advantaged Janajati	575 (17.9)	179 (15)	238 (19.1)	117 (19.5)	41 (23)	
	Dalit/Religious Minorities/Others	457 (14.2)	233 (19.6)	152 (12.2)	52 (8.7)	20 (11.2)	
**Marriage**	Single	539 (16.7)	448 (37.6)	48 (3.8)	26 (4.3)	17 (9.5)	<0.001
	Currently Married	2680 (83.3)	743 (62.4)	1201 (96.2)	575 (95.7)	161 (90.5)	
**Occupation**	Unemployed	175 (5.4)	60 (5)	58 (4.6)	35 (5.8)	22 (12.4)	<0.001
	Employed (Govt/ Non-govt)	379 (11.8)	101 (8.5)	172 (13.8)	91 (15.1)	15 (8.4)	
	Self-employed	840 (26.1)	167 (14)	412 (33)	209 (34.8)	52 (29.2)	
	Home Makers	1450 (45)	497 (41.7)	600 (48)	266 (44.3)	87 (48.9)	
	Student	375 (11.6)	366 (30.7)	7 (0.6)	0	2 (1.1)	
**Family size**	1-4 members	2007 (62.3)	685 (57.5)	816 (65.3)	400 (66.6)	106 (59.5)	<0.001
	≥5 members	1212 (37.6)	506 (42.5)	433 (34.7)	201 (33.4)	72 (40.5)	
**Household Wealth Quintile**	Lowest	644 (20.0)	257 (21.6)	233 (18.7)	125 (20.8)	29 (16.3)	0.004
	Second	643 (20.0)	233 (19.6)	246 (19.7)	124 (20.6)	40 (22.5)	
	Middle	645 (20.0)	249 (20.9)	248 (19.9)	107 (17.8)	41 (23.0)	
	Fourth	643 (20.0)	235 (19.7)	251 (20.1)	120 (20.0)	37 (20.8)	
	Highest	644 (20.0)	217 (18.2)	271 (21.7)	125 (20.8)	31 (17.4)	
**Region**	East (Koshi)	506 (15.7)	151 (12.7)	224 (17.9)	107 (17.8)	24 (13.5)	<0.001
	Middle (Bagamati/Gandaki)	1038 (32.2)	295 (24.8)	451 (36.1)	226 (37.6)	66 (37.1)	
	South (Madhesh/Lumbini)	803 (24.9)	292 (24.5)	302 (24.2)	157 (26.1)	52 (29.2)	
	West (Karnali/Sudur Paschim)	872 (27.1)	453 (38)	272 (21.8)	111 (18.5)	36 (20.2)	
**Residence**	Urban	2085 (64.8)	735 (61.7)	855 (68.5)	386 (64.2)	109 (61.2)	0.004
	Rural	1134 (35.2)	456 (38.3)	394 (31.5)	215 (35.8)	69 (38.8)	
**Any kind of Tobacco Use**	Yes	857 (26.6)	166 (13.9)	339 (27.1)	263 (43.8)	89 (50)	<0.001
	No	2362 (73.4)	1025 (86.1)	910 (72.9)	338 (56.2)	89 (50)	
**Drinking Alcohol**	Yes	928 (28.8)	366 (30.7)	347 (27.8)	159 (26.5)	56 (31.5)	0.171
	No	2291 (71.2)	825 (69.3)	902 (72.2)	442 (73.5)	122 (68.5)	
**Physical Activity**	Insufficient	607 (18.9)	221 (18.6)	200 (16)	132 (22)	54 (30.3)	<0.001
	Sufficient	2612 (81.1)	970 (81.4)	1049 (84)	469 (78)	124 (69.7)	
**Any kind of Stress**	Yes	2421 (75.2)	808 (67.8)	1001 (80.1)	481 (80)	131 (73.6)	<0.001
	No	798 (24.8)	383 (32.2)	248 (19.9)	120 (20)	47 (36.4)	
**BMI**	Normal	1991 (61.8)	814 (68.4)	728 (53.3)	339 (56.4)	110 (61.8)	<0.001
	Overweight	777 (24.1)	181 (15.2)	377 (30.2)	180 (29.9)	39 (21.9)	
	Obesity	194 (6)	30 (2.5)	100 (8)	54 (9)	10 (5.6)	
	Underweight	257 (8)	166 (13.9)	44 (3.5)	28 (4.7)	19 (10.7)	
**Hypertension**	Yes	882 (27.4)	169 (14.2)	330 (26.4)	281 (46.8)	102 (57.3)	<0.001
	No	2337 (72.6)	1022 (85.8)	919 (73.6)	320 (53.2)	76 (42.7)	
**Hypercholesterolemia**	Yes	325 (10.1)	60 (5)	151 (12.1)	95 (15.8)	19 (10.7)	<0.001
	No	2894 (89.9)	1131 (95)	1098 (87.9)	506 (84.2)	159 (89.3)	
**Diabetes**	Yes	208 (6.5)	26 (2.2)	78 (6.2)	80 (13.3)	24 (13.5)	<0.001
	No	3011 (93.5)	1165 (97.8)	1171 (93.8)	521 (86.7)	154 (86.5)	

**Note:** Values are presented as n (%), unless otherwise specified. Age is presented as mean ± standard deviation (SD). P-values were calculated using Chi-square tests for categorical variables. Statistical significance was defined as p < 0.05. BMI = body mass index; SD = standard deviation.

Prevalence estimates for behavioral, metabolic, and clinical risk factors and NCDs were calculated within each age group using the number of participants with the respective condition divided by the total number of participants in that age category (Table 1). Overall prevalence of hypertension, diabetes, and hypercholesterolemia in the study population was 27.4%, 6.5%, and 10.1%, respectively. Tobacco use and physical inactivity were highest among participants aged ≥60 years, whereas stress prevalence was highest among the 30–44 and 45–59 years age groups. Overweight and obesity were more common among middle-aged adults, particularly those aged 30–44 and 45–59 years. Hypertension and diabetes prevalence were highest among participants aged ≥60 years (57.3% and 13.5%, respectively), while hypercholesterolemia prevalence was highest among those aged 45–59 years (15.8%). Significant differences across age groups were observed for all variables except alcohol consumption.

### Association of sociodemographic and risk factors with NCDs

Table 2 presents the multivariable logistic regression analysis of risk factors associated with hypertension, diabetes, and hypercholesterolemia. Participants aged ≥60 years had the highest odds of hypertension (aOR: 4.92, 95% CI: 3.35–7.22), while those aged 45–59 years had the highest odds of diabetes (aOR: 6.34, 95% CI: 3.58–11.23) and hypercholesterolemia (aOR: 2.66, 95% CI: 1.77–3.98) compared with participants aged 15–29 years. Male participants had significantly higher odds of hypertension than females (aOR: 2.04, 95% CI: 1.62–2.55) Participants with bachelor-level education had higher odds of diabetes compared with those with less than secondary education (aOR: 2.09, 95% CI: 1.12–3.89). Compared with upper caste groups, disadvantaged ethnic groups showed higher diabetes (aOR 1.51: 95%CI: 1.04–2.20), whereas relatively advantaged Janajati groups had lower odds of hypercholesterolemia (aOR 0.49 95%CI: 0.34–0.72). Participants from the South region had lower odds of hypertension (aOR:0.69, 95%CI: 0.53–0.92), but higher odds of diabetes (aOR:1.69, 95%CI: 1.07–2.67), compared with those from the East region. Rural residence was associated with lower diabetes risk (aOR: 0.43, 95% CI: 0.29–0.63). Tobacco use was associated with increased odds of hypertension (aOR=1.29, 95% CI: 1.04–1.6), while insufficient physical activity (aOR:1.49, 95%CI: 1.06–2.12) and stress (aOR:1.53, 95%CI: 1.04–2.25) were associated with higher odds of diabetes (p < 0.05). Overweight and obesity were consistently associated with increased odds of hypertension and hypercholesterolemia, with obesity showing the strongest associations (hypertension aOR=3.50, 95%CI: 2.51–4.88; hypercholesterolemia aOR=2.91, 95%CI: 1.97–4.29). Underweight status was inversely associated with hypertension risk (aOR=0.61, 95%CI: 0.41–0.91). Marital status and occupation showed no significant associations. The multivariable logistic regression models demonstrated adequate goodness of fit. The Hosmer–Lemeshow test was non-significant for the hypertension model (χ² = 3.94, df = 8, *p* = 0.863), diabetes model (χ² = 12.95, df = 8, *p* = 0.114), and hypercholesterolemia model (χ² = 5.49, df = 8, *p* = 0.704), indicating no evidence of poor model fit. The corresponding Nagelkerke R² values were 0.215, 0.181, and 0.092, respectively, indicating modest explanatory power, with the hypertension model showing the highest explanatory power. No substantial multicollinearity was detected among the predictors (all VIF values <5 and tolerance values >0.1).

**Table 2:** Multivariable logistic regression analysis of factors associated with hypertension, diabetes, and hypercholesterolemia (N = 3219).

Variables	Categories	Hypertension		Diabetes		Hypercholesterolemia	
		aOR (95%CI)	p-value	aOR (95%CI)	p-value	aOR (95%CI)	p-value
**Age Group (years)**	15-29	1		1		1	
	30-44	1.44 (1.13-1.84)	**<0.01**	2.57 (1.49-4.44)	**<0.001**	1.90 (1.32-2.72)	**<0.001**
	45-59	3.05 (2.33-4.02)	**<0.001**	6.34 (3.58-11.23)	**<0.001**	2.66 (1.77-3.98)	**<0.001**
	≥60	4.92 (3.35-7.22)	**<0.001**	6.29 (3.15-12.56)	**<0.001**	1.97 (1.09-3.59)	**<0.05**
**Sex**	Female	1		1		1	
	Male	2.04 (1.62-2.55)	**<0.01**	0.93 (0.62-1.4)	0.76	0.72 (0.52-1.00)	**<0.05**
**Education**	Less than secondary	1		1		1	
	Secondary	0.85 (0.71-1.03)	0.09	0.88 (0.62-1.23)	0.45	1.01 (0.78-1.32)	0.93
	Above Secondary	1.03 (0.68-1.56)	0.88	2.09 (1.12-3.89)	**<0.05**	1.24 (0.72-2.15)	0.43
**Ethnicity**	Upper caste	1		1		1	
	Disadvantaged Janajati/Terai)	1.18(0.94-1.48)	0.16	1.51 (1.04-2.20)	**<0.05**	0.86 (0.63-1.17)	0.34
	Advantaged Janajati	1.09 (0.85-1.4)	0.49	0.66 (0.39-1.1)	0.11	0.49 (0.34-0.72)	**<0.001**
	Others	1.72 (1.31-2.25)	**<0.001**	1.46 (0.92-2.31)	0.11	0.86 (0.59-1.27)	0.46
**Marriage**	Single	1		1		1	
	Currently Married	1.03 (0.73-1.4)	0.88	1.58 (0.75-3.33)	0.23	1.01 (0.61-1.67)	0.97
**Occupation**	Unemployed	1		1		1	
	Employed (Govt/Non-govt)	1.17 (0.76-1.79)	0.47	0.53 (0.28-1.02)	0.06	1.26 (0.68-2.33)	0.46
	Self-employed	1.15 (0.78-1.69)	0.48	0.43 (0.24-0.77)	**<0.01**	1.07 (0.61-1.88)	0.82
	Home Makers	0.99 (0.67-1.46)	0.96	0.44 (0.24-0.79)	**<0.01**	1.02 (0.58-1.79)	0.95
	Student	0.66 (0.37-1.16)	0.15	0.79 (0.27-2.25)	0.65	0.83 (0.35-1.94)	0.66
**Family size**	1-4 family members	1		1		1	
	≥5 family members	1.06 (0.88-1.28)	0.50	2.09 (1.53-2.85)	**<0.01**	1.21 (0.94-1.57)	0.14
**Region**	East (Koshi)	1		1		1	
	Middle (Bagamati/Gandaki)	0.88 (0.68-1.14)	0.32	0.71 (0.43-1.17)	0.18	0.81 (0.57-1.14)	0.22
	South Terai (Madhesh/Lumbini)	0.69 (0.53-0.92)	**<0.05**	1.69 (1.07-2.67)	**<0.05**	0.78 (0.54-1.12)	0.18
	West (Karnali/ Sudur Paschim)	0.82 (0.61-1.09)	0.17	0.73 (0.42-1.26)	0.26	0.47 (0.31-0.72)	**<0.001**
**Residence**	Urban	1		1		1	
	Rural	0.97 (0.81-1.17)	0.76	0.43 (0.29-0.63)	**<0.001**	0.81 (0.62-1.06)	0.12
**Any kind of Tobacco**	No	1		1		1	
	Yes	1.29 (1.04-1.6)	**<0.05**	1.31 (0.90-1.89)	0.16	1.28 (0.93-1.77)	0.13
**Physical Activity**	Sufficient	1		1		1	
	Insufficient	1.07 (0.85-1.33)	0.57	1.49 (1.06-2.12)	**<0.05**	0.95 (0.7-1.30)	0.77
**Any kind of Stress**	No	1		1		1	
	Yes	1.10 (0.89-1.35)	0.38	1.53 (1.04-2.25)	**<0.05**	0.93 (0.70-1.22)	0.59
**BMI**	Normal	1		1		1	
	Overweight	1.87 (1.53-2.29)	**<0.001**	1.09 (0.76-1.54)	0.64	1.44 (1.09-1.89)	**<0.05**
	Obesity	3.50 (2.51-4.88)	**<0.001**	1.65 (0.96-2.82)	0.07	2.91 (1.97-4.29)	**<0.001**
	Underweight	0.61 (0.41-0.91)	**<0.05**	0.47 (0.21-1.05)	0.06	0.75 (0.41-1.36)	0.34
**Model fitness**							
Hosmer–Lemeshow χ² (df)		3.94 (8)		12.95 (8)		5.49 (8)	
Hosmer–Lemeshow χ² (df); p-value		0.863		0,114		0.704	
Nagelkerke R²		0.215		0.181		0.092	

**Note:** Values are presented as adjusted odds ratios (aORs) with 95% confidence intervals (CI) derived from multivariable logistic regression analyses. BMI = body mass index. Model goodness of fit was assessed using the Hosmer–Lemeshow test, and Nagelkerke R^2^ was reported as a measure of pseudo-R^2^. Model fit was additionally evaluated using the likelihood ratio test and Akaike information criterion (AIC). Receiver operating characteristic (ROC) analysis was used to assess model discrimination. No significant multicollinearity was detected (all VIF values <5 and tolerance values >0.1).

### Association of age groups with NCDs and related risk factors

Unadjusted logistic regression analyses showed increasing odds of NCDs with advancing age (S3 Table). Participants aged ≥60 years had the highest odds of hypertension (OR=8.12, 95% CI: 5.78–11.39, p < 0.001) and diabetes (OR=6.98, 95%CI: 3.91–12.47 p < 0.001), while hypercholesterolemia was highest among those aged 45–59 years (OR=3.54, 95% CI: 2.52–4.97, p < 0.001) compared with participants aged 15–29 years.

Behavioral and metabolic risk factors also varied across age groups. Tobacco use and physical inactivity were most prevalent among older adults aged ≥60 (OR=6.17 and 1.91, respectively), whereas stress was more common among middle-aged adults (OR 1,91 for aged 30–44 and 1.90 for 45–59 years). BMI patterns revealed lower odds of normal weight status in middle-aged groups (OR: 0.78 and 0.72 for age groups 30–44 and 45–59 years) compared to 15–29 years (Table 3).

**Table 3:** Unadjusted logistic regression analysis of association between age group with hypertension, diabetes and hypercholesterolemia; and selected risk factors (N = 3219).

[A]: NCDs												
Age Group (years)	Hypertension				Diabetes				Hypercholesterolemia			
	OR	95% CI		p-value	OR	95% CI		p-value	OR	95% CI		p-value
15-29	1				1				1			
30-44	2.17	1.77	2.67	**< 0.001**	2.98	1.90	4.69	**< 0.001**	2.59	1.9	3.53	**< 0.001**
45-59	5.31	4.23	6.67	**< 0.001**	6.88	4.37	10.84	**< 0.001**	3.54	2.52	4.97	**< 0.001**
≥60	8.12	5.78	11.39	**< 0.001**	6.98	3.91	12.47	**< 0.001**	2.25	1.31	3.87	**< 0.01**
**[B]: Selected Risk factors**												
**Age Group (years)**	**Tobacco consumption**							**Insufficient Physical Activity**				
	**OR**	**95% CI**			**p-value**			**OR**	**95% CI**			**p-value**
15-29	1							1				
30-44	2.3	1.87		2.83	**< 0.001**			0.84	0.68	1.03		0.09
45-59	4.80	3.82		6.05	**< 0.001**			1.23	0.97	1.57		0.09
≥60	6.17	4.41		8.64	**< 0.001**			1.91	1.34	2.72		**< 0.001**
**Age Group (years)**	**Perceived any Stress**							**BMI**				
	**OR**	**95% CI**			**p-value**			**OR**	**95% CI**			**p-value**
15-29	1							1				
30-44	1.91	1.59		2.30	**< 0.001**			0.78	0.67	0.92		**< 0.01**
45-59	1.9	1.50		2.40	**< 0.001**			0.72	0.59	0.87		**< 0.001**
≥60	1.32	0.93		1.88	0.12			0.79	0.58	1.09		0.16

**Note:** Values are presented as crude odds ratios (OR) with 95% confidence intervals (CI). Reference categories are indicated as “1”. Statistical significance was considered at *p* < 0.05.

The nonparametric Wilcoxon and Kruskal Wallis test was chosen for right-skewed data in normality tests displayed in normal distribution plot (S1 Fig*.,* S2 Table). Significant differences in the distribution of hypertension, diabetes, and hypercholesterolemia across age groups were observed in Wilcoxon rank-sum analyses (Fig 3; S3 Table**.**).

**Fig 3:**
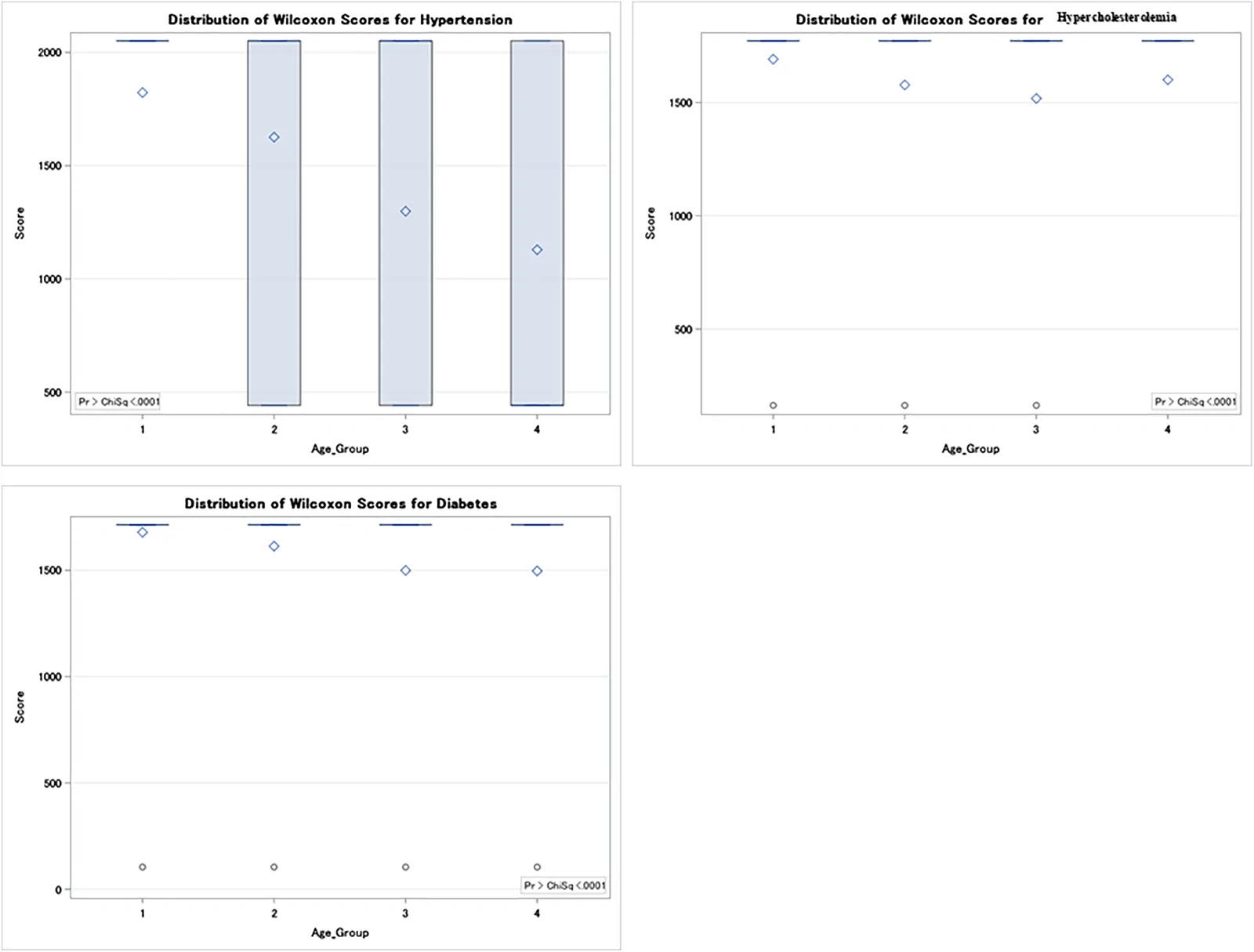
Wilcoxon Scores (Rank Sums) for NCDs among stratified age groups (Kruskal Wallis Test).

S3 Table summarizes the distributions of hypertension, diabetes, and hyperlipidemia across the four age groups. Although median values showed limited variation because the NCD variables were binary, their distributions differed significantly across age groups for hypertension (χ^2^ = 298.19, *p* < 0.001), diabetes (χ^2^ = 97.31, *p* < 0.001), and hyperlipidemia (χ^2^ = 60.68, *p* < 0.001), based on the Kruskal–Wallis test.

Post-hoc pairwise comparisons using the Dwass–Steel–Critchlow–Fligner method demonstrated the largest differences between the 15–29 and 45–59 years age groups (Table 4). Additional significant differences were observed between the 30–44 and 45–59 years groups for hypertension and diabetes, whereas differences among older age groups were smaller or non-significant. Hypercholesterolemia showed significant increases from young adulthood to middle age, with comparatively smaller differences thereafter.

**Table 4:** Post-hoc Dwass-Steel-Critchlow-Fligner (DSCF) pairwise comparisons of hypertension, diabetes and hypercholesterolemia distributions across age groups (N = 3219).

Comparison of age group (Years)	Hypertension			Diabetes			Hypercholesterolemia		
	Wilcoxon Z	DSCF Value	p-value	Wilcoxon Z	DSCF Value	p-value	Wilcoxon Z	DSCF Value	p-value
15-29 vs. 30–44	7.5	10.6	**<0.001**	5.0	7.0	**<0.001**	6.2	8.8	**<0.001**
15-29 vs. 45–59	15.0	21.2	**<0.001**	9.4	13.3	**<0.001**	7.7	10.8	**<0.001**
15-29 vs. ≥ 60	13.5	19.0	**<0.001**	7.5	10.6	**<0.001**	3.0	4.3	**<0.05**
30-44 vs. 45–59	8.7	12.3	**<0.001**	5.1	7.2	**<0.001**	2.2	3.1	0.12
30-44 vs. ≥ 60	8.4	11.9	**<0.001**	3.5	5.0	**<0.01**	−0.5	0.8	0.95
45-59 vs. ≥ 60	2.5	3.5	0.06	0.1	0.1	0.99	−1.7	2.4	0.32

**Note:** P-values were derived from DSCF post-hoc pairwise comparisons following Kruskal–Wallis tests. Statistical significance was considered at *p* < 0.05.

ROC curves analyses demonstrated moderate discriminatory ability of age for hypertension, diabetes, and hypercholesterolemia (Fig 4). The area under the curve (AUC) values were 0.700 for hypertension and 0.698 for diabetes, and 0.627 for hypercholesterolemia, indicating statistically significant predictive ability for all three conditions (p < 0.001).

**Fig 4:**
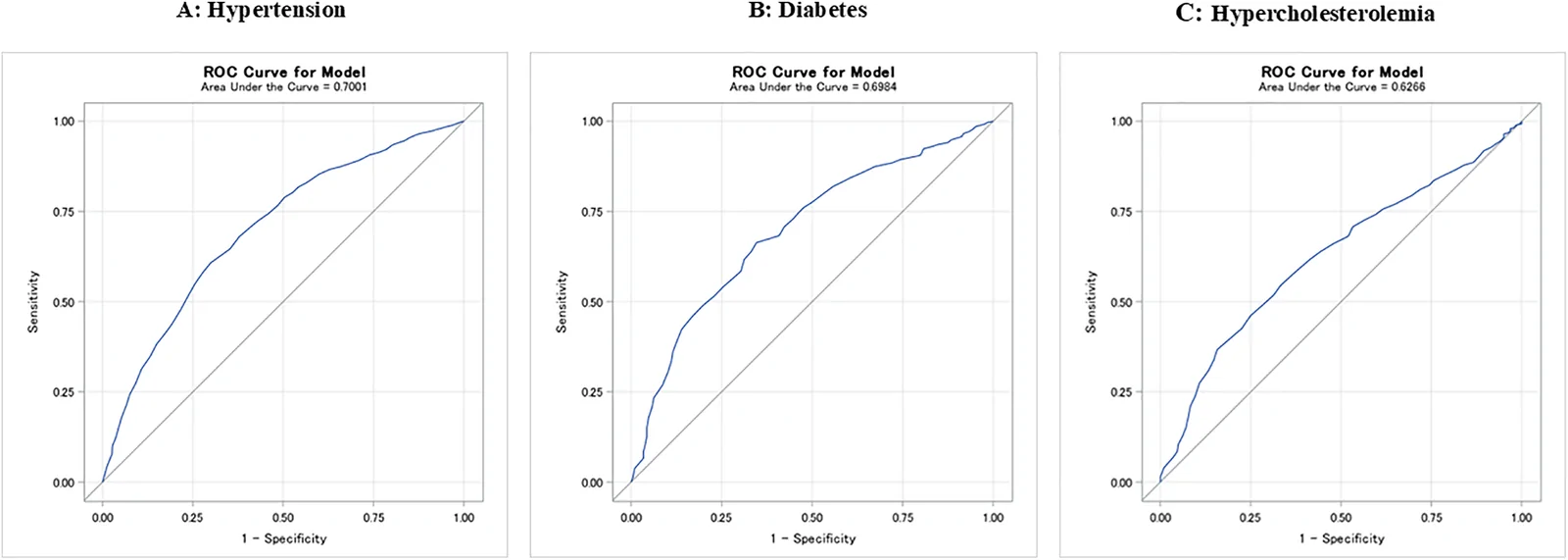
Receiver Operating Characteristics (ROC) analysis by age for NCDs prediction.

## Discussion

### Prevalence of NCDs/risk factors

Previous studies indicated tobacco and alcohol are primary risk factors of NCDs in elderly [20–26]. Our study found similar results Half of the older adults used tobacco. Still, young adults also have notable prevalence of risk factors:14% using tobacco, 30.7% alcohol consumption after older adults (31.5%), indicating a notable NCDs risk. The increasing burden of NCDs across age groups may reflect Nepal’s ongoing epidemiological and nutritional transition, characterized by urbanization, sedentary lifestyles, unhealthy dietary patterns, tobacco use, and reduced physical activity [2,4,13,27]. Similar trends have been reported across South Asian countries undergoing rapid demographic and lifestyle changes.

A pooled analysis of 358 population-based surveys (2001–2016) showed South Asia had the second highest rate of physical inactivity (33%) globally [28]. In comparison to this result, our study found it lower (19%), with the highest in older adults (30.3%) and second highest in young adults (15–29 years) (18.6%), which indicated significant risk among young adults that was higher than middle aged adults (30–44 and 45–60 years). Adults >18 years lived with obesity rose from 7% to 16% during 1990–2022, while in children and adolescents aged 5–19 grew from 2% to 8% globally [29]. We found higher obesity and overweight prevalence in middle-aged-adults (30–44 and 45–59 years). Although the prevalence of overweight is lower among younger adults compared to other age groups, approximately 15% of young adults are still overweight, highlighting a concerning risk for non-communicable diseases (NCDs) at an early age. Similar findings among young adults and adolescents have been reported in previous global and South Asian studies [13,14,27]. These findings suggest that NCD prevention strategies in Nepal should begin earlier in life rather than focusing predominantly on older [13,14,27]. Older adults exhibited the highest prevalence of diabetes (13.5%) and hypertension, while middle-aged adults (aged 45–59 years) had the highest rates of hypercholesterolemia. Notably, among young adults, 14% had diabetes, 2% had hypertension, and 5% had hypercholesterolemia, underscoring a significant non-communicable disease (NCD) burden even at a younger age.

### Association of age with NCDs/risk factors

Studies from Nepal and India showed higher NCDs prevalence among older adults [28,29]. Our results consistently demonstrated a strong association between aging and NCDs prevalence. Similar age-related increases in NCD prevalence and associated behavioral risk factors have also been reported in other South Asian populations, highlighting the growing regional burden of chronic diseases linked to demographic and lifestyle transitions [14,20,21,30]. However, upper-middle-aged adults (45–59 years) had a higher association of hypercholesterolemia and diabetes than older adults. Previous studies among young adults/adolescents showed similar outcomes and projections [13,27]. Univariate-logistic regression had similar results with diabetes and hypercholesterolemia, but the highest hypertension risk in older adults. Tobacco consumption, obesity/overweight and insufficient physical activity were most common among older adults, while stress was highest among 45–60 years. Young adults had the highest Wilcoxon-Scores for hypertension and hypercholesterolemia, and 30–44 for diabetes, indicating significant NCD burden in young adults. Pairwise comparisons showed significant differences in NCD risk across age groups. The ROC curve suggested moderate accuracy in predicting NCD risk by age.

### Association of sociodemographic/risk factors with NCDs

Like a US study [31], men had twice the risk of hypertension, but no gender differences in diabetes or hypercholesterolemia. The Disadvantaged Janajati had a higher diabetes risk, possibly due to marginalization and low awareness, while Relatively Advantaged had lower hypercholesterolemia risk compared to upper caste. Surprisingly, individuals with graduate-level education had double diabetes risk compared to those with lesser education; further in-depth study in this issue is needed. The South region, with the lowest HDI [31], had higher diabetes risk but lower hypertension risk and West had lower hypercholesterolemia risk, compared to East. Urban residents had a higher diabetes risk, possibly due to sedentary lifestyle and unhealthy eating habits such as more processed junk food; more research is needed. A study in aged ≥50 in China and Ghana showed wealth-related health disparities with NCD morbidity [30], however, we could not found significant association between household wealth quintile or marital status and NCDs. A recent study from Nepal reported an increased risk of hypertension and diabetes among government employees [32]. However, our analysis did not reveal a statistically significant association between governmental/private jobs and NCDs.

Tobacco increased hypertension risk, while stress and insufficient exercise raised diabetes risk. These associations are biologically plausible, as obesity, physical inactivity, and stress contribute to insulin resistance, vascular dysfunction, chronic inflammation, and metabolic dysregulation [22,33–35]. This aligns with a Swedish study [33] that found higher stress-related-disease diagnosed (OR: 3.35) with diabetes. As previous articles [34,35] suggested obesity/overweight were associated with higher hypertension, diabetes, and hypercholesterolemia prevalence, we found obese and overweight individuals had higher hypercholesterolemia and hypertension risk.

These findings highlight the importance of age-specific prevention strategies, including behavioral interventions targeting younger populations, early screening among middle-aged adults, and continuity of care for older adults. Strengthening primary healthcare-based NCD screening and health promotion programs may support Nepal’s progress toward reducing premature NCD-related morbidity and mortality [2,36].

Although this study identified significant associations between age and major NCDs, interaction/effect-modification analyses were not performed because the primary objective was to assess overall age-stratified associations at the population level. Future studies should explore whether these associations differ across subgroups such as sex, residence, and socioeconomic status to support more targeted prevention strategies.

### Strengths and limitations

Use of nationally representative data from the WHO-standard STEPS survey is one of the strengths of this study. Still, there are limitations. Cross-sectional study design limited causal inferences. Self-reported responses on alcohol consumption, tobacco use, physical inactivity, and stress might be biased due to fear of criticism. Some important risk factors, such as unhealthy eating habits, could not be included in the analysis due to insufficient data availability for these variables. The data lacked information on ≥70 years adults and frequency/amount of alcohol and tobacco use, which could impact on NCD prevalence. In addition, interaction or effect-modification analyses were not conducted; therefore, potential differential effects of risk factors across subgroups such as sex, residence, and socioeconomic status could not be evaluated. Further studies incorporating interaction analyses may provide deeper understanding of subgroup-specific NCD risk patterns.

## Conclusion

This study demonstrated significant age-related differences in the prevalence of hypertension, diabetes, hypercholesterolemia, and associated behavioral and metabolic risk factors in Nepal. Although NCD prevalence increased with age, substantial behavioral and metabolic risks were also observed among younger adults, indicating early exposure to modifiable risk factors. These findings highlight the importance of age-specific prevention strategies, including health promotion and behavioral interventions for younger populations, early screening and risk-factor management among middle-aged adults, and strengthened continuity of care for older adults. Integrating age-stratified NCD prevention and screening programs into primary healthcare systems may support Nepal’s progress toward achieving Sustainable Development Goal target 3.4 on reducing premature NCD-related morbidity and mortality.

### Policy implication

The findings of this study highlight the need for age-specific and life-course approaches to NCD prevention and control in Nepal. The notable prevalence of behavioral and metabolic risk factors among younger adults suggests that prevention strategies should begin earlier in life through school-, community- and primary healthcare-based health promotion interventions focusing on tobacco control, harmful alcohol reduction, healthy diet, and physical activity promotion.

Middle-aged adults require strengthened screening and early detection programs targeting hypertension, diabetes, obesity, and hypercholesterolemia to prevent progression and complications. For older adults, improving continuity of care, medication adherence, and access to long-term NCD management services is essential.

The findings also support strengthening primary healthcare-based NCD prevention and screening programs aligned with Nepal’s Multisectoral Action Plan for NCDs and Sustainable Development Goal target 3.4 to reduce premature NCD-related morbidity and mortality. Integrated multisectoral strategies addressing lifestyle, behavioral, and metabolic risk factors across the life course are necessary to reduce the growing NCD burden in Nepal [37,38].

## Supporting information

S1 File Dataset_STEPS Survey Nepal_2019. Dataset used in the present study, including the variables used for the analysis of age-stratified prevalence and associated risk factors of hypertension, diabetes, and hypercholesterolemia.
(XLSX)

S1 Table Operational definitions of variables. Operational definitions, measurement criteria, and categorization of the sociodemographic, behavioral, biomedical, and clinical variables included in the analysis.
(DOCX)

S1 Fig Normal Distribution Plot. Graphical assessment of the distribution of age used to evaluate the normality of the continuous variable and inform the selection of appropriate statistical methods.
(DOCX)

S2 Table Normality test for age distribution. Results of the statistical assessment of the normality of age distribution, including the relevant test statistics and p-values.
(DOCX)

S3 Table Comparison of NCD distributions across age groups. Statistical comparison of the distributions of hypertension, diabetes, and hypercholesterolemia across the four age groups, including the results of rank-based statistical tests.
(DOCX)
