# Supplementary material for: Age-stratified prevalence and associated risk factors of hypertension, diabetes and hypercholesterolemia in Nepal: Insights from the STEPS survey 2019
Source: PLoS One. 2026 Oct 16;21(10):e0340932. doi: 10.1371/journal.pone.0340932 (PMC13674751; doi:10.1371/journal.pone.0340932)
Supplement: Supplementary file 1 [file pone.0340932.s002.docx]

**S1 Table. Operational definitions of variables**

| Variables | Operational definitions |
| --- | --- |
| Hypertension | Having average systolic and/or diastolic blood pressure readings of 140 mmHg and/or 90 mmHg respectively after three measurements, and/or currently on hypertension medication as recommended by the World Health Organization (17). |
| Diabetes | Having fasting blood glucose >125 mg/dl and/or currently under treatment according to the American Diabetes Association's guidelines (18) |
| Hypercholesterolemia | Having a total cholesterol level exceeding 200 mg/dl and/or taking medication for Hypercholesterolemia (19). |
| Tobacco use | Currently using any kind of tobacco |
| Alcohol consumption | Currently using alcohol (consumed within past 12 months) |
| Insufficient physical activity | < 75 minutes of vigorous physical activity or <150 minutes of moderate physical activity per week |
| Any stress | Perceived any kind of stress caused by work/business stress or general stress at home or severe financial stress/due to unemployment or stressful life events in past year which disturbed a lot |
