# Supplementary material for: Age-stratified prevalence and associated risk factors of hypertension, diabetes and hypercholesterolemia in Nepal: Insights from the STEPS survey 2019
Source: PLoS One. 2026 Oct 16;21(10):e0340932. doi: 10.1371/journal.pone.0340932 (PMC13674751; doi:10.1371/journal.pone.0340932)
Supplement: Supplementary file 3 [file pone.0340932.s004.docx]

**S2 Table. Normality test for age distribution**

**Test for location**

| Test | Statistic | | p Value | |
| --- | --- | --- | --- | --- |
| Student's t | t | 156.4679 | Pr > \|t\| | <0.001 |
| Sign | M | 1609.5 | Pr ≥ \|M\| | <0.001 |
| Signed Rank | S | 2591295 | Pr ≥ \|S\| | <0.001 |

**Test for normality**

| Test | Statistic | | p Value | |
| --- | --- | --- | --- | --- |
| Kolmogorov-Smirnov | D | 0.074872 | Pr > D | <0.010 |
| Cramer-von Mises | W-Sq | 4.375913 | Pr > W-Sq | <0.005 |
| Anderson-Darling | A-Sq | 29.2708 | Pr > A-Sq | <0.005 |
