# Supplementary material for: Age-stratified prevalence and associated risk factors of hypertension, diabetes and hypercholesterolemia in Nepal: Insights from the STEPS survey 2019
Source: PLoS One. 2026 Oct 16;21(10):e0340932. doi: 10.1371/journal.pone.0340932 (PMC13674751; doi:10.1371/journal.pone.0340932)
Supplement: Supplementary file 4 [file pone.0340932.s005.docx]

**S3 Table. Comparison of NCD distributions across age groups using Wilcoxon rank scores and Kruskal–Wallis Tests (N=3219)**

| **Age Group (Years)** | **n** | **Hypertension (p<0.001)** | | | | **Diabetes (p<0.001)** | | | | **Hypercholesterolemia (p<0.001)** | | | |
| --- | --- | --- | --- | --- | --- | --- | --- | --- | --- | --- | --- | --- | --- |
|  |  | **Sum of Scores** | **Expected Under H_0_** | **SD Under H_0_** | **Mean Rank** | **Sum of Scores** | **Expected Under H_0_** | **SD Under H_0_** | **Mean Rank** | **Sum of Scores** | **Expected Under H_0_** | **SD Under H_0_** | **Mean Rank** |
| 15-29 | 1191 | 2170735.5 | 1917510.0 | 19666.7 | **1822.6** | 1999527.0 | 1917510.0 | 10840.6 | **1678.9** | 2014477.5 | 1917510.0 | 13284.9 | **1691.4** |
| 30-44 | 1249 | 2030564.0 | 2010890.0 | 19849.7 | **1625.8** | 2015245.0 | 2010890.0 | 10941.5 | **1613.5** | 1970818.0 | 2010890.0 | 13408.6 | **1577.9** |
| 45-59 | 601 | 780381.5 | 967610.0 | 15873.1 | **1298.5** | 901354.0 | 967610.0 | 8749.6 | **1499.8** | 912370.0 | 967610.0 | 10722.4 | **1518.1** |
| ≥60 | 178 | 200909.0 | 286580.0 | 9310.2 | **1128.7** | 266464.0 | 286580.0 | 5131.9 | **1497.0** | 284924.5 | 286580.0 | 6289.1 | **1600.7** |
| Kruskal-Wallis p-vlue |  | <0.001 | | | | <0.001 | | | | <0.001 | | | |

*Note: Values are Wilcoxon rank scores, with average ranks assigned to tied observations. The mean rank is the average Wilcoxon rank score within each age group. Overall differences in the distributions of the NCD outcomes across age groups were assessed using the Kruskal–Wallis test. When the overall test was statistically significant, pairwise comparisons were performed using the Dwass-Steel-Critchlow-Fligner (DSCF) method based on Wilcoxon rank sums. All tests were two-sided, with p < 0.05 considered statistically significant. H₀, null hypothesis; SD, standard deviation.*
