# Supplementary material for: Age-stratified prevalence and associated risk factors of hypertension, diabetes and hypercholesterolemia in Nepal: Insights from the STEPS survey 2019
Source: PLoS One. 2026 Oct 16;21(10):e0340932. doi: 10.1371/journal.pone.0340932 (PMC13674751; doi:10.1371/journal.pone.0340932)
Supplement: Supplementary file 5 [file pone.0340932.s003.docx]

**S1 Fig.** **Normal Distribution Plot**
